# Non-Ligation of Umbilical Cord

**Published:** 1915-06

**Authors:** 


**Non-Ligation of Umbilical Cord.** A. L. Rachmanow, Zentralblatt fuer Gyn. 1914, reports 10,000 cases. The cord is cut 4 c.m. from the umbilicus, after the vessels cease to pulsate, that is about 12-20 minutes after delivery of the foetus. In cases of haemophilia, syphilis, and immaturity, amounting to about 17%, ligation was deemed advisable or was required. No deaths from haemorrhage of the unligated cord occurred.

